# Supplementary material for: Ataxin‐3 consolidates the MDC1‐dependent DNA double‐strand break response by counteracting the SUMO‐targeted ubiquitin ligase RNF4
Source: EMBO J. 2017 Mar 8;36(8):1066–83. doi: 10.15252/embj.201695151 (PMC5391139; doi:10.15252/embj.201695151)
Supplement: Supplementary file 1 — Appendix [file EMBJ-36-1066-s001.pdf]

APPENDIX

Table of Contents

Appendix Supplementary Figure Legends Page 2-4

Appendix Supplementary Figures S1-S9 Page 5-13

Appendix Supplementary Materials and Methods Page 14-20

Supplementary References Page 21

Appendix Figure Legends

Appendix Figure S1. Mutation of the VCP-binding motif in ataxin-3 prevents it from binding to VCP/p97 and recruitment of VCP to DSBs is independent of ataxin-3. (**A**) U2OS cells were transiently transfected with GFP or GFP-ataxin3 wt, C14A, *UIMs, *VBM or with the GFP-tagged Josephin domain of ataxin-3. Cells were lysed in co-IP buffer and GFP containing complexes were purified on GFP-trap agarose. Immunoblotting was performed with VCP/p97, GFP and β-actin antibodies. (**B**) Laser micro-irradiation was performed in U2OS cells depleted of ataxin-3 by two independent siRNAs. Cells were fixed at 1 h post-irradiation, immunostained for VCP/p97 and analyzed by immunofluorescence. The relative increase of VCP/p97 at DSBs was quantified. Data are presented as mean ± SEM of three independent experiments.

Scale bars: 5μm.

Appendix Figure S2. Knock-down efficiency of Ubc9 and PIAS4 and ataxin-3/SUMO1 interaction. (**A**) Depletion of Ubc9 and PIAS4 by specific Ubc9- and PIAS4-targeting siRNAs. (**B**) *In vitro* SUMO binding assay. Recombinant ataxin-3 was incubated with unconjugated agarose beads or with recombinant SUMO1 immobilized on agarose beads. Binding of ataxin-3 to recombinant SUMO1 was analyzed by immunoblotting.

Appendix Figure S3. Depletion efficiency of endogenous ataxin-3 by two different ataxin-3 siRNAs and effect on steady-state levels of various DDR proteins. (**A**) Knock-down efficiencies of ataxin-3 in U2OS upon transfection with siATX3-1 and siATX3-2. As a loading control β-actin is shown. (**B**) Steady-state levels of DNA damage response proteins upon depletion of ataxin-3 were analyzed by immunoblotting with indicated antibodies.

Appendix Figure S4. Ataxin-3 and MDC1 interact in a SUMOylation-independent fashion and the recruitment of ataxin-3 to DSBs is not dependent on MDC1. (**A**) U2OS cells stably expressing GFP-ataxin-3 were depleted of PIAS4 or Ubc9. Cells

were exposed to DNA damage induction (bleomycin) and co-immunoprecipitation was performed on endogenous MDC1 (or IgG). Immunoblotting was performed with indicated antibodies. (*) indicates an unspecific band that runs lower than GFP-ataxin-3. (**B**) Co-immunoprecipitation was performed on GFP or GFP-ataxin-3 expressing U2OS cells transfected with HA-tagged wild-type MDC1 or MDC1 K1840R under DNA damaging conditions. Immunoblotting was performed with indicated antibodies. (**C**) U2OS-DSB reporter cells were transfected with control or MDC1-specific siRNA and transiently transfected with GFP-ataxin-3. DSBs were induced by stabilizing the LacR-FokI fusion protein and cells were analyzed by immunolabeling. The depletion efficiency of MDC1 was tested by immunoblotting. Note also that the MDC1 immunofluorescent signal was drastically reduced following MDC1 depletion.

Scale bar: 5μm.

Appendix Figure S5. Quantification approach of protein accumulation at laser-induced DNA damage lines. Fluorescent levels at laser-inflicted DNA damage lines were determined using an unbiased, semi-automated quantification approach using the Volocity software (PerkinElmer). Microscopy images of γH2AX immunostaining and Hoechst staining were used for segmentation of the DNA damage and the nucleus area, respectively. Fluorescence intensities in these segmentations were measured and a relative enrichment of proteins of interest at DSBs was calculated by taking a ratio of the mean intensity in the DNA damage area versus the mean intensity in the no-DNA damage area.

Appendix Figure S6. Ataxin-3 stimulates the recruitment of BRCA1 and 53BP1 to IRIF. (**A**) Control or ataxin-3-depleted U2OS cells were exposed to ionizing radiation, examined by immunofluorescence and the number of BRCA1 IRIF was quantified (**B**) As in (A) but the number of 53BP1 IRIF was analyzed and quantified. Data are presented as mean ± SEM of three independent experiments. **** $P \leq 0.0001$ (Kruskal-Wallis test).

Scale bars: 5 μm.

Appendix Figure S7. Ataxin-3 mediates the recruitment of 53BP1 to DSBs in a dose-dependent manner. (**A**) Parental U2OS and U2OS cells stably expressing GFP-ataxin-3 or GFP-ataxin-3^{C14A} were depleted of ataxin-3 by two different siRNAs. Laser micro-irradiation was conducted, cells were fixed at 1 h post laser damage and immunostained for 53BP1 and γH2AX. (**B**) Knock-down efficiencies of endogenous and/or ectopically expressed ataxin-3 in parental U2OS and U2OS cells stably expressing GFP-ataxin-3 or GFP-ataxin-3^{C14A}. (**C**) Parental U2OS or U2OS cells stably expressing GFP-ataxin-3 were subjected to laser damage and immunostained for 53BP1. The relative intensity of 53BP1 at sites of laser damage was quantified. Data are presented as mean ± SEM of two independent experiments.

Scale bars: 5 μm.

Appendix Figure S8. Knock-down efficiencies in ataxin-3-, RNF4- or double-depleted cells. U2OS cells transfected with specific ataxin-3, RNF4 or both siRNAs were efficiently depleted of the respective proteins.

Appendix Figure S9. The depletion of RNF4 has mild effect on the recruitment of XRCC4 and cells are sensitized to ionizing radiation in the absence of ataxin-3. (**A**) U2OS cells were transfected with control or RNF4 directed siRNA, subjected to laser micro-irradiation and immunostained for XRCC4 and γH2AX. The relative increase of XRCC4 at laser-induced DNA damage was quantified. Data are presented as mean ± SEM of three independent experiments. * $P \leq 0.05$ (Mann-Whitney test). (**B**) For survival experiments, VH10-SV40 cells were transfected with indicated siRNAs, seeded at low density and exposed to the indicated doses of ionizing radiation. Cells were incubated for 7 days and stained with methylene blue. Colonies of more than 10 cells were scored. Data are presented as mean ± SEM of two independent experiments.

Scale bar: 5 μm.

Appendix Figure S1

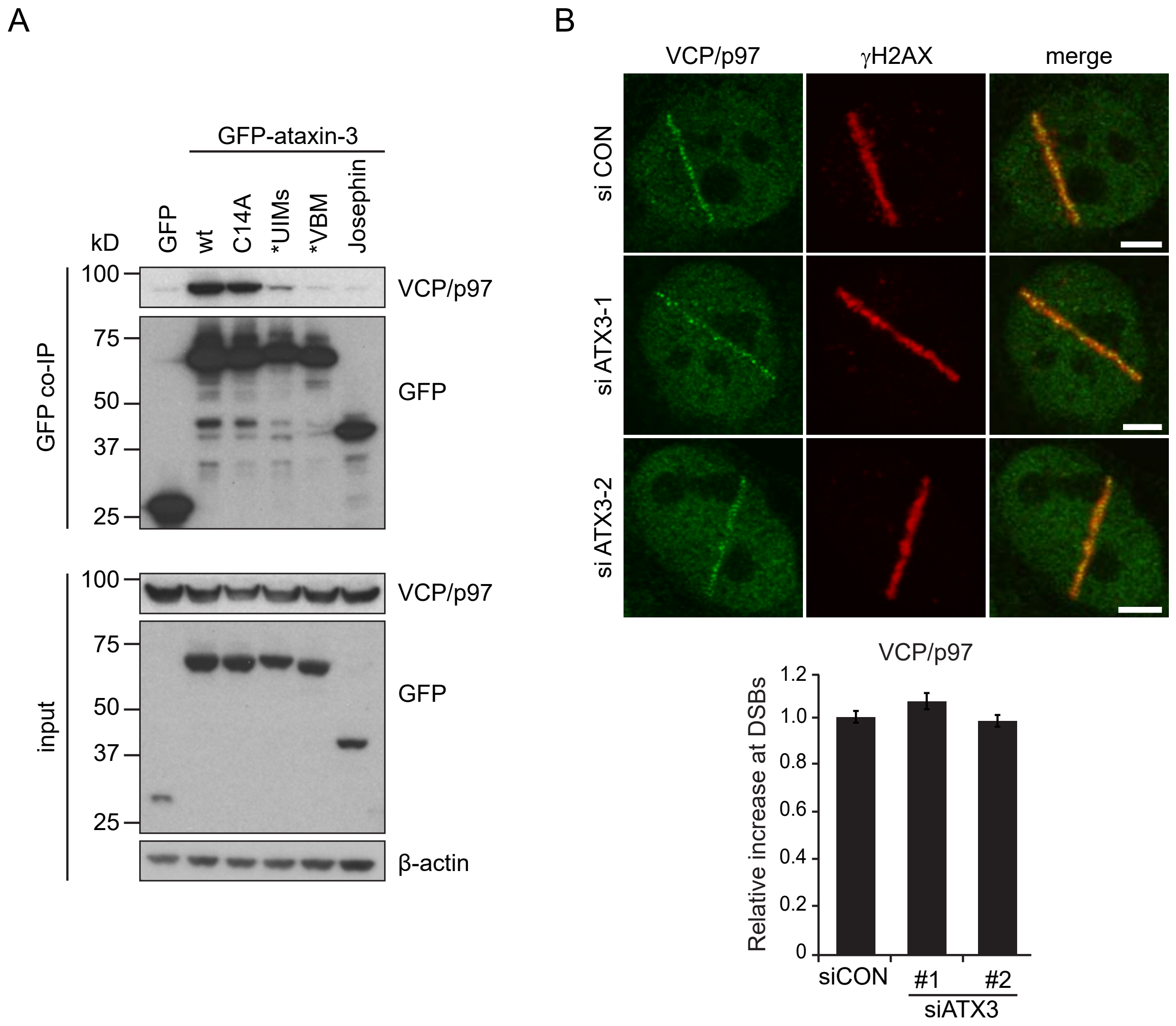

Appendix Figure S2

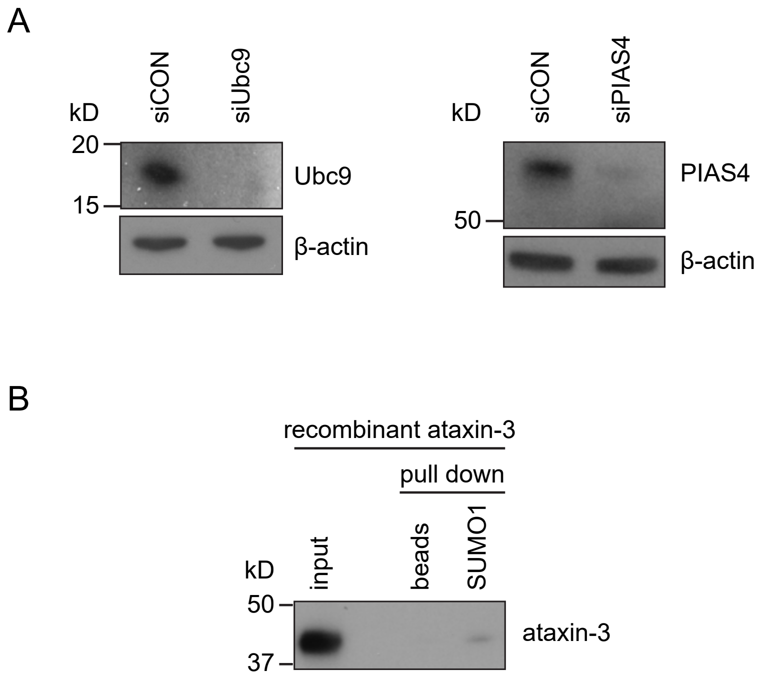

Appendix Figure S3

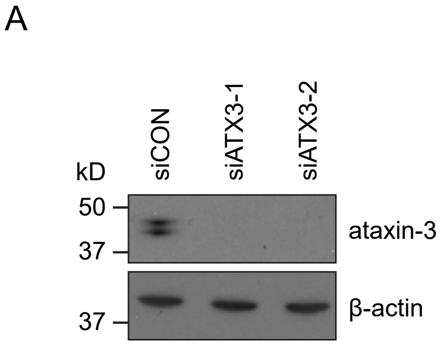

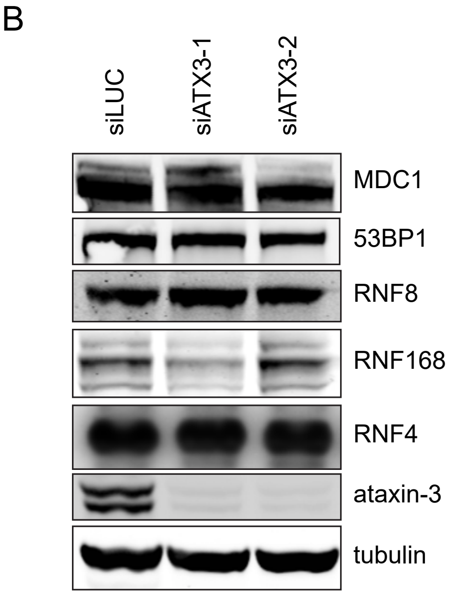

Appendix Figure S4

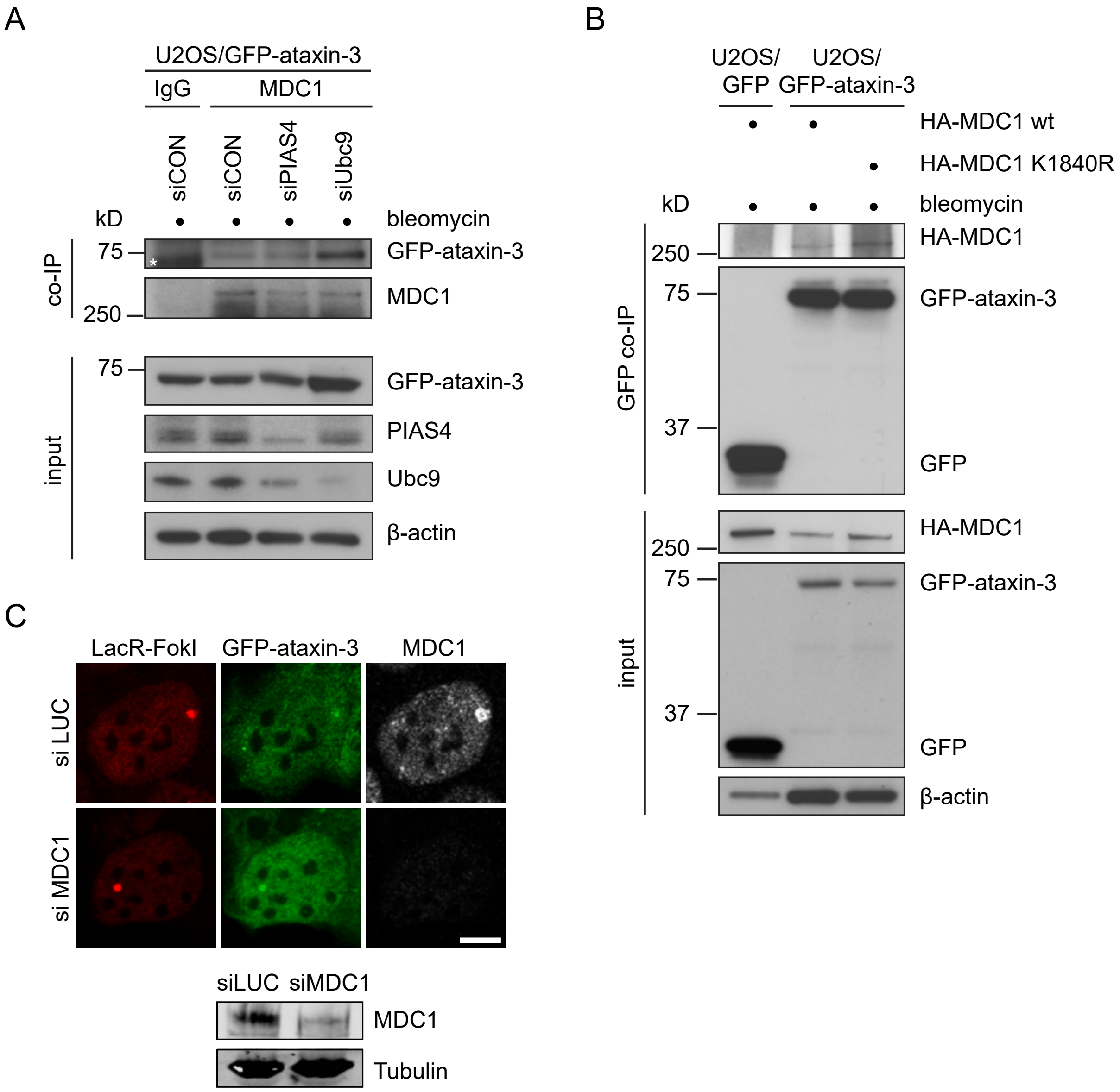

Appendix Figure S5

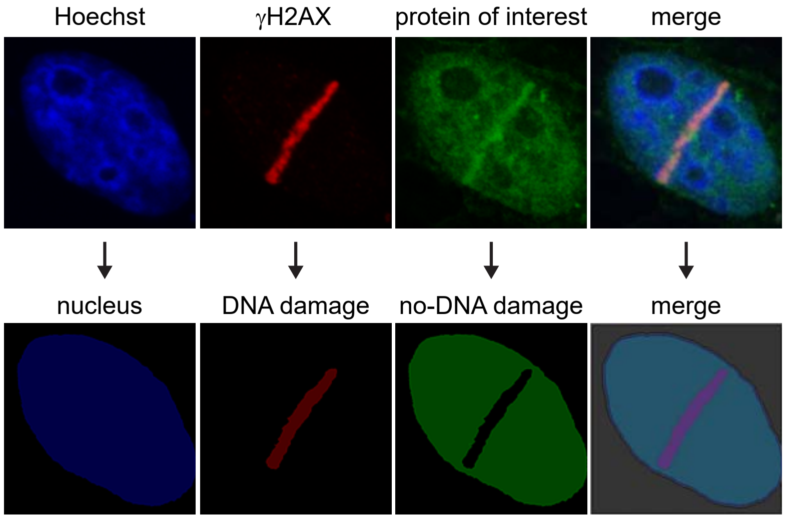

relative intensity of protein of interest (POI) at DSBs =Appe

$$\frac{\text{mean POI “DNA damage”}}{\text{mean POI “no-DNA damage”}}$$

Appendix Figure S6

A

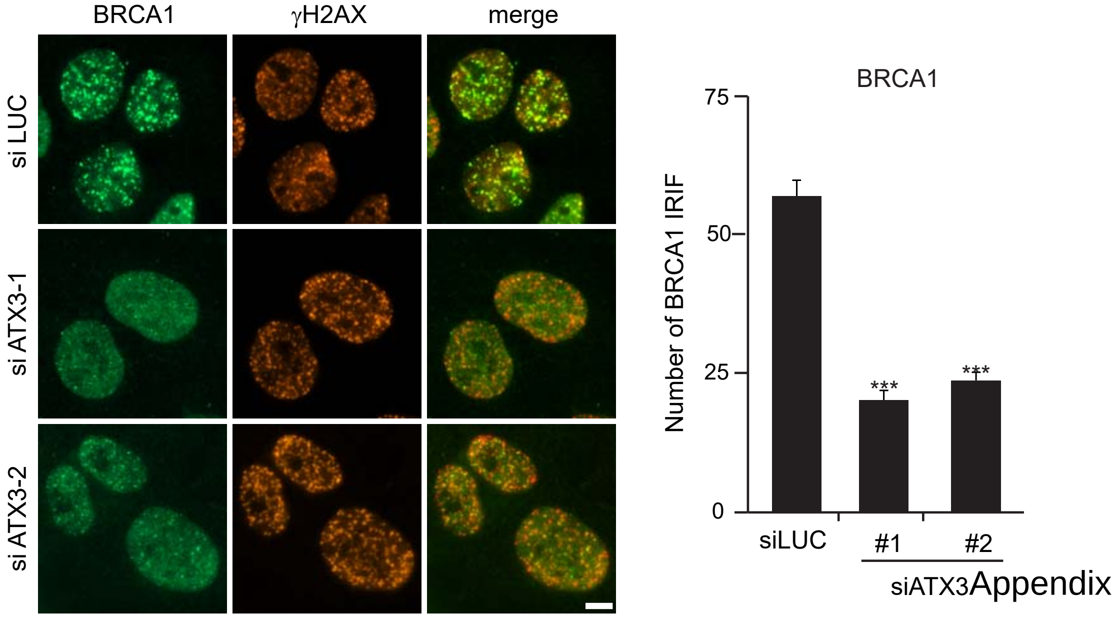

B

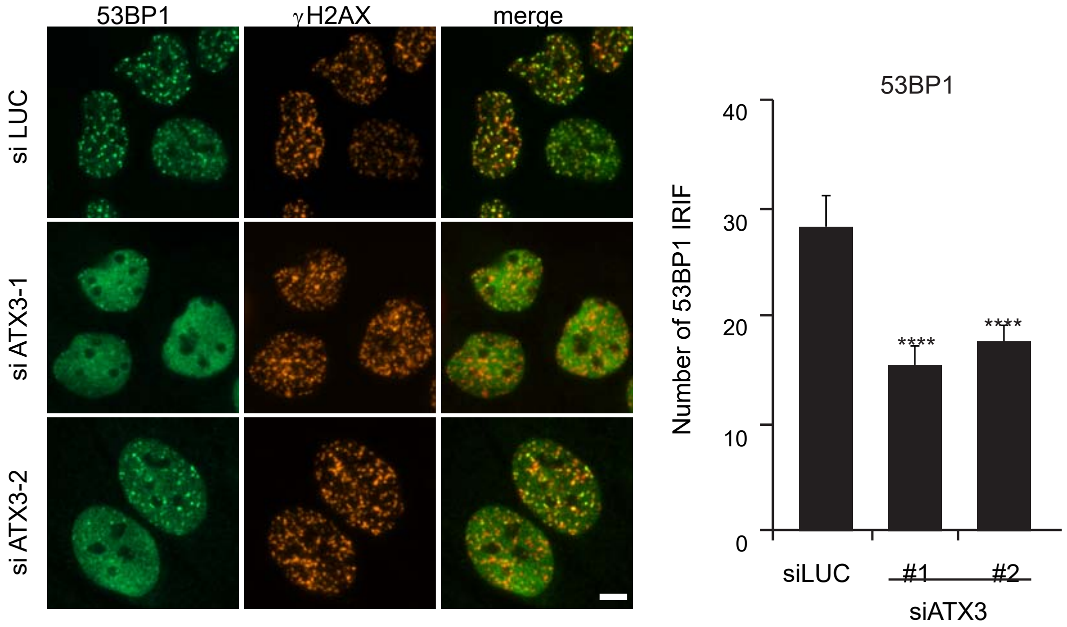

Appendix Figure S7

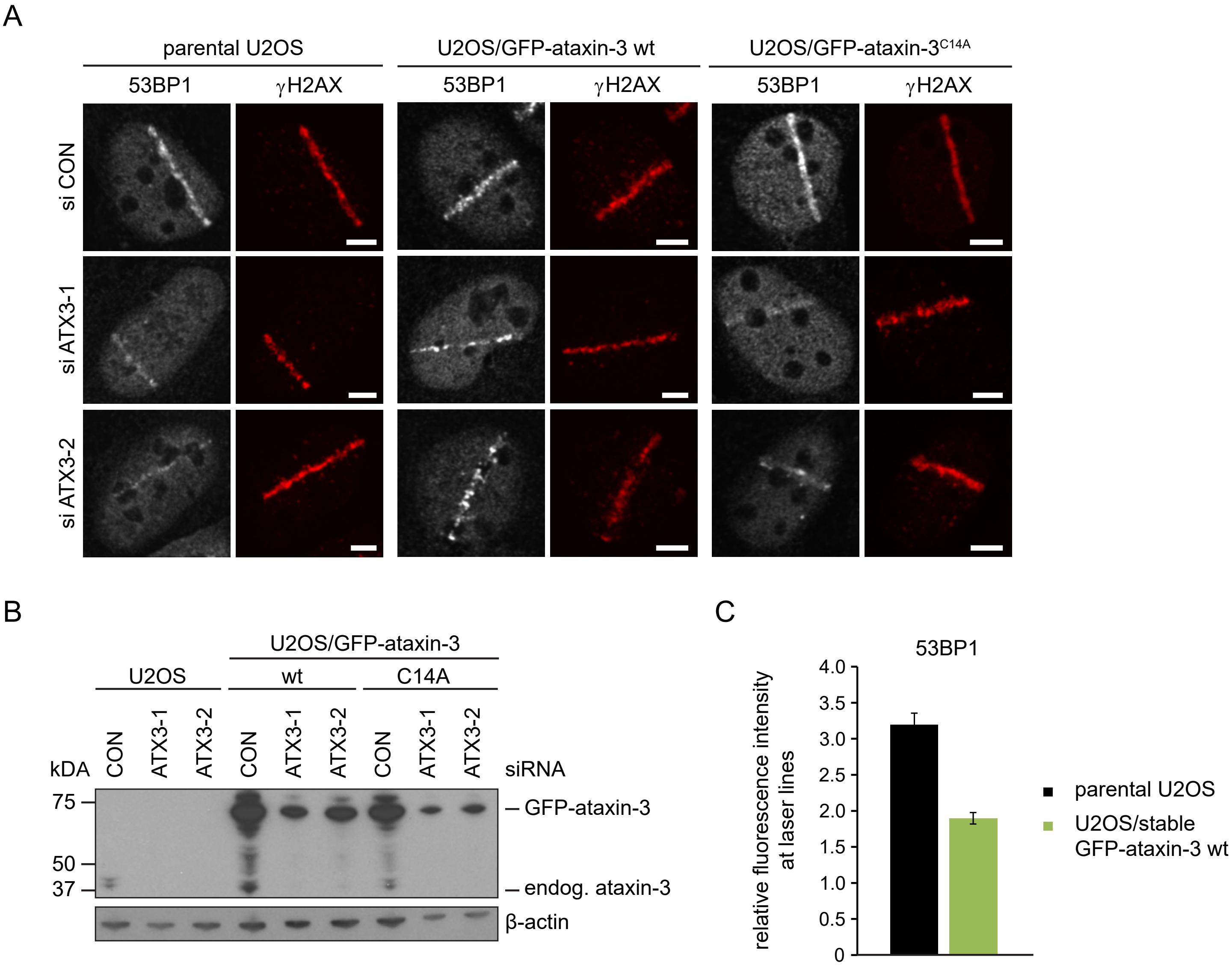

Appendix Figure S8

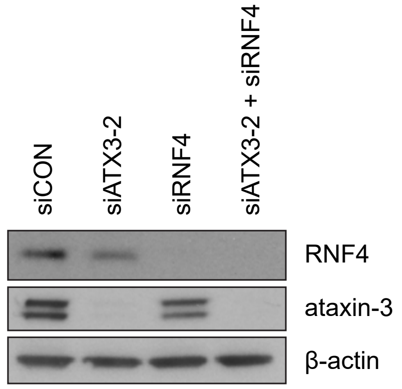

Appendix Figure S9

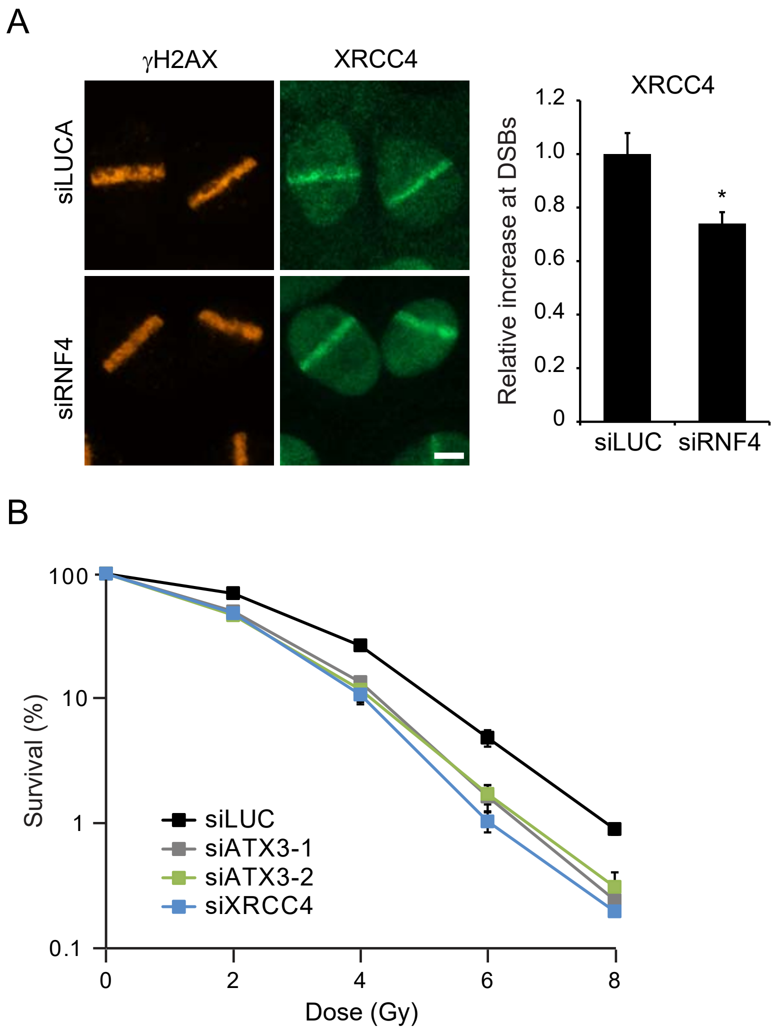

Supplementary Materials and Methods

Cell culture

Cells were cultured in DMEM GlutaMAX (Life Technologies) supplemented with 10% fetal bovine serum (FBS) and antibiotics at 37°C in a humidified chamber with 5% CO_2. pTuner265 DSB reporter cells (stably expressing ER-mCherry-LacR-FokI-DD (Tang et al, 2013)) were induced for 5 h by 1 μM Shield-1 (Clontech) and 1 μM 4-OHT. Stable U2OS cell lines expressing GFP-ataxin-3 or GFP-ataxin-3^{C14A} were generated by transfection of parental U2OS cells with pEGFP-ataxin-3-IRES-Puro and the pEGFP-ataxin-3^{C14A}-IRES-Puro plasmids, respectively. After 24 h, cells expressing the GFP-fusion protein were selected by replacing the culture medium with medium containing 1 μg/ml puromycin. Clones expressing the GFP-ataxin-3 fusion protein were isolated and validated by fluorescence microscopy and Western blotting. To generate HeLa TET-on inducible cell lines stably expressing 10xHis-ataxin-3-HA or 10xHis-ataxin-3^{C14A}-HA C14A, HeLa Flp-In T-REx cells were co-transfected with pOG44 (encoding the Flp recombinase) and pcDNA5/FRT/TO-Flp-In-10xHis-ataxin-3-HA or pcDNA5/FRT/TO-Flp-In-10xHis-ataxin-3^{C14A}-HA and at 48 h post-transfection selection by replacing the culture medium with medium containing 100 μg/ml hygromycin B was started. Expression was induced by addition of 1 μg/ml doxycycline for 24 h. Clones expressing 10xHis-ataxin-3-HA or 10xHis-ataxin-3^{C14A}-HA were isolated and validated by Western blotting. Other cell lines used in this study were parental U2OS cells, stable GFP-MDC1 cells (Galanty et al, 2012), and HEK293 cells containing a stably integrated copy of the DR-GFP (Pierce et al, 1999) or EJ5-GFP (Bennardo et al, 2008) reporter for HR or NHEJ, respectively. U2OS cells stably expressing doxycycline-inducible shRNF8 and catalytic inactive Flag-RNF8*RING were induced by the addition of doxycycline for 72 h (Mailand et al, 2007) (gift from Niels Mailand, University of Copenhagen). To induce DSBs, 10 μg/ml bleomycin sulfate (Enzo Life Science) or 20 μM camptothecin (Sigma) were added to cells for 1 h.

Plasmids

GFP-ataxin-3 was generated by PCR-amplifying ataxin-3 from YFP-ataxin-3 (gift from Harm H. Kampinga; University of Groningen) and ligating into an EGFP-C1 vector (Clontech) by using the *Xho*I/*Bam*HI restriction sites. To create catalytic inactive ataxin-

3^{C14A}, site-directed mutagenesis (QuickChange, Stratagene) was performed using GFP-ataxin-3 as template to exchange two nucleotides using the primers 5'-AAACAAGAAGGCTCACTTGCTGCTCAACATTGCCTGAATAAC-3' and 5'-GTTATTCAGGCAATGTTGAGCAGCAAGTGAGCCTTCTTGTTTC-3'. The GFP-ataxin-3^{UIM*} plasmid was sub-cloned by PCR amplification of the mutant ataxin-3 open reading frame from FLAG-ataxin-3^{UIM*} (gift from Henry Paulson, University of Michigan; (Todi et al, 2009)), which was inserted into EGFP-C1 (Clontech). GFP-ataxin-3^{VBM*} was obtained by inserting mutations in the VCP-binding motif ^{282}RKRR to ^{282}HNHH (Boeddrich et al, 2006). Based on consensus sequences (V/I/L-x-V/I/L-V/I/L or V/I/L-V/I/L-x-V/I/L where x is any amino acid) for SUMO-interacting motifs (SIM), a SIM was identified in ataxin-3 as ^{162}IFVV. By a two-step overlapping PCR the SIM was mutated from ^{162}IFVV to ^{162}AFAA. PCR amplified ataxin-3^{SIM*} was inserted into EGFP-C1 by using *Xho*I/*Bam*HI restriction sites. To obtain pcDNA5/FRT/TO-10xHis-ataxin-3-HA, ataxin-3 was inserted into pcDNA5/FRT/TO with a generated C-terminal HA tag. An N-terminal 10xHis tag was introduced using a double-stranded oligo. To create pcDNA5/FRT/TO-10xHis-ataxin-3^{SIM*}-HA, ataxin-3^{SIM*} was PCR amplified from the GFP-ataxin-3^{SIM*} vector and ligated into pcDNA5/FRT/TO-10xHis-HA using *Kpn*I/*Bam*HI restriction sites. An IRES-Puro cassette was inserted into *Eco*RV site of pEGFP-C1 to generate pEGFP-C1-IRES-puro. The ATXN3 cDNA was sub-cloned from FLAG-HA-ataxin-3 (Addgene: 22538; (Sowa et al, 2009)) into pEGFP-IRES-Puro using *Bsr*GI sites. pEGFP-ataxin-3^{C14A}-IRES-puro plasmid was generated by sub-cloning the N-terminal sequence of ataxin-3 C14A from the pEGFP-ataxin-3^{C14A} vector into the pEGFP-ataxin-3-IRES-puro vector using *Age*I and *Pst*I restriction sites. GST-ataxin-3 was obtained by PCR amplifying ataxin-3 from the GFP-ataxin-3 construct and inserting it into a pGEX-6P-1 vector. Cloning results were verified by sequencing (GATC Biotech). Expression plasmids for GFP-RNF4 (gift from Alfred Vertegaal; Leiden University Medical Center), GFP-SUMO1 (gift from Ron Hay, University of Dundee), GFP-Ubc9 (gift from Jin-Hyun Ahn, Sungkyunkwan University School of Medicine), HA-MDC1 and HA-MDC1 K1840R (gift from Zhenkun Lou, Mayo Clinic) were used.

Transfections

Plasmid DNA transfections were performed using Lipofectamine 2000 (Life Technologies). The siRNA transfections were performed using Lipofectamine

RNAiMAX (Life Technologies) or Lipofectamine 2000 according to the manufacturer's instructions. siRNA oligonucleotides were purchased from GE healthcare/Dharmacon.

RNA interference

The following siRNA sequences were used in this study: AllStars negative control siRNA (Qiagen); ATX3 #1, 5'-GCA CUA AGU CGC CAA GAA A-3'; ATX3 #2, 5'-ACG AAG AUG AGG AGG AUU U-3'; Ubc9, 5'-CAA AAA AUC CCG AUG GCA C-3'; PIAS4, 5'-GGA GUA AGA GUG GAC UGA A-3'; RNF4, 5'-GAA UGG ACG UCU CAU CGU U-3'; XRCC4, 5'-AUA UGU UGG UGA ACU GAG A-3'; LUC, 5'-CGU ACG CGG AAU ACU UCG A-3', KU80 5'-CAA GGA UGA GAU UGC UUU AGU-3', MDC1 5'-GTC TCC CAG AAG ACA GTG A-3'; BRCA2, 5'- GAA GAA UGC AGG UUU AAU A-3'.

Immunofluorescent labeling

Immunofluorescent labeling was performed as previously described (Acs et al., 2011). Primary antibodies and dilutions were rabbit anti-53BP1 1:200 (Novus Biologicals, NB100-304), mouse anti-BRCA 1:100 (Santa Cruz, D-9 sc-6954), mouse anti-conjugated ubiquitin (FK2) 1:100 (Enzo Life Science, BML-PW8810), rabbit anti-MDC1 1:1000 (Abcam, ab11169), mouse anti-γH2AX 1:1000 (Millipore, JBW301), rabbit anti-γH2AX 1:500 (Cell Signaling, 20E3), mouse anti-VCP/p97 1:1000 (Abcam, ab11433), rabbit anti-ataxin-3 1:250 (gift from Thorsten Schmidt, University of Tübingen), rabbit anti-RNF168 1:200 (Millipore, ABE367), mouse anti-RNF8 1:20 (Santa Cruz, B-2 sc-271462), anti-XRCC4 (gift from Mauro Modesti, Cancer Research Center of Marseille, CNRS), mouse anti-RPA32 (Abcam, ab2175), rabbit anti-RAD51 (Santa Cruz, H-92 sc-8349).

Western blotting

Cell extracts were boiled in LDS sample buffer supplemented with 300 mM DTT, separated by SDS-PAGE (4-12% NuPAGE Bis-Tris protein gel, Life Technologies) and transferred to PVDF membrane (Millipore). Membranes were blocked in Tris-buffered saline/5% skimmed milk. Immunoblotting was performed with the following antibodies: mouse anti-ataxin-3 1:1000 (Millipore, 1H9 MAB5360), mouse anti-VCP/p97 1:5000 (Abcam, ab11433), mouse anti-β-actin 1:5000 (Sigma-Aldrich, A2228), rabbit anti-GFP 1:5000 (Abcam, ab290), mouse anti-MDC1 1:1000 (Sigma, M2444), rabbit anti-MDC1 1:1500 (Abcam, ab11169), mouse anti-HA 1:1000

(Covance, MMS-101R), rabbit anti-Ubc9 1:1000 (Cell Signaling, #4918), rabbit anti-PIAS4 1:1000 (Cell Signaling, D2F12 #4392), rabbit anti-RNF4 1:500 (gift from Jorma Palvimo, University of Eastern Finland), chicken anti-RNF4 1:5000 (gift from Ron Hay, University of Dundee), rabbit anti-ubiquitin 1:1000 (Cell Signaling, #3933), mouse anti-His 1:2000 (MBL International, JM-3646), mouse anti-tubulin 1:2000 (Sigma, T6199), rabbit anti-53BP1 1:2000 (Novus Biologicals, NB100-304), mouse anti-RNF8 1:1000 (Santa Cruz, B-2 sc-271462), rabbit anti-RNF168 1:500 (Millipore, ABE367). Secondary antibodies used were HRP-linked sheep anti-mouse IgG (GE Healthcare, NXA931V) or donkey anti-rabbit IgG (GE Healthcare, NA934V) 1:5000 followed by ECL detection on film (Fuji).

Cell survival assay

For survival experiments, VH10-SV40 cells were transfected with siRNAs, trypsinized, seeded at low density and either exposed to IR or to different concentrations of PARPi (KU0058948). Cells were subsequently incubated for 7 days, washed with 0.9% NaCl and stained with methylene blue. Colonies of more than 10 cells were scored.

Microscopy analysis

Images of fixed cells were acquired on a Zeiss AxioImager D2 widefield fluorescence microscope equipped with 40x, 63x and 100x PLAN APO (1.4 NA) oil-immersion objectives (Zeiss) and an HXP 120 metal-halide lamp used for excitation or on a Zeiss LSM510 META confocal microscope equipped with a 63x Plan-A (1.4 NA) oil-immersion lens. Images were recorded using ZEN 2012 software or Zeiss LSM imaging software in multi-track mode. Images were analyzed using ImageJ or Zeiss LSM image browser. The average reflects the quantification of 50-150 cells from 2-3 independent experiments.

Statistical analysis

Statistical analysis was performed using GraphPad Prism 6. To test for a Gaussian data distribution, the Shapiro-Wilk normality test was used. If the normality test was passed, data were analysed by Student’s unpaired t-test (two groups) or by one-way Anova test (more than two groups). If the data were not normally distributed, statistical analysis was performed using the nonparametric Mann-Whitney test (two groups) or Kruskal-Wallis test for multiple comparisons. Data are shown as mean from at least 2-

3 independent experiments. Error bars represent the ±SEM (standard error of the mean). The following P values were considered significant * P≤0.05; ** P≤0.01; *** P≤ 0.001; **** P≤0.0001.

Immunoprecipitation

Denaturing MDC1 immunoprecipitation. Cells were transfected with indicated siRNAs (48 h). At 24 h post-transfection, cells were treated for 1 h with 10 µg/ml bleomycin to introduce DSBs. Cells were harvested in ice-cold 1x phosphate buffer saline (PBS) and resuspended in 200 µl denaturing RIPA buffer supplemented with 1% benzonase (Millipore) (50 mM Tris pH 7.5, 400 mM NaCl, 1 mM EDTA, 5 mM $MgCl_2$, 1% NP-40, 1% sodium deoxycholate, 0.1% SDS, 20 mM NEM, 10 µM MG132, 1x complete EDTA-free protease inhibitor (Roche)). After an incubation time of 30 min on ice, insoluble debris was pelleted at 16.100 g 10 min at 4°C. An aliquot of the supernatant was saved as input sample before diluting the lysate x5 with RIPA buffer not containing detergent to a final NaCl concentration of 150 mM (10 min on ice). To the lysate 50 µl dynabeads protein G (Life technologies) coupled to polyclonal rabbit MDC1 antibody (Bethyl, A300-053A) or rabbit IgG (Abcam, ab46540) was added and incubated for 1.5 h at 4°C under constant rotation. The beads were washed six times (2x 150 mM NaCl, 2x 500 mM NaCl, 2x 150 mM NaCl) with washing buffer (50 mM Tris pH 7.4, 150 mM NaCl, 1 mM EDTA, 5 mM $MgCl_2$, 20mM NEM, 10 µM MG132, 1x complete EDTA-free protease inhibitor, 0.1% NP-40) and eluted with 2x LDS sample buffer containing reducing agent.

For co-immunoprecipitation on MDC1, cells were treated or not with 10 µg/ml bleomycin to induce DNA damage. Cells were harvested in ice-cold 1x PBS (500 g 3 min 4°C). Cells were resuspended in 1 ml Triton extraction buffer (PBS containing 0.5% Triton X-100, supplemented with 20 mM NEM, 10 µM MG132, 1x complete EDTA-free protease inhibitor) and nuclei were pelleted at 2000 rpm 5 min at 4°C. The nuclei pellet was resuspended in 200 µl nuclear buffer containing 50 mM Tris pH 7.5, 1 mM EDTA, 0.27 M sucrose, 20 mM NEM, 10 µM MG132, 1 mM β-glycerolphosphate, 1 mM Na_3VO_4, 1x complete EDTA-free protease inhibitor, supplemented with 1% benzonase, sonicated and insoluble debris was pelleted at 16.100 g 10 min at 4°C. The supernatant was pre-cleared with 10 µl dynabeads protein G (Life Technologies) for 30 min at 4°C under constant rotation. After pre-clearing, the nuclear lysate was

incubated with 50 µl dynabeads protein G conjugated to rabbit polyclonal MDC1 antibody (Bethyl, A300-053A) or rabbit IgG (Abcam, ab46540) for 1.5 h at 4°C under constant rotation. The agarose beads were washed six times with nuclear buffer and eluted with 2x LDS sample buffer.

For purification of GFP containing complexes, cells transiently transfected with GFP-fusion expression vectors or stably expressing GFP fusion proteins were harvested in ice-cold 1x PBS (500 g 3 min 4°C). Where indicated, DNA damage was induced by 10 µg/ml bleomycin for 1 h prior to harvesting cells. The cell pellet was resuspended in 200 µl ice-cold wash buffer (10 mM Tris pH 7.5, 150 mM NaCl, 0.5 mM EDTA, 20 mM NEM, 2 mM MgCl2, 10 µM MG132, 1x complete EDTA-free protease inhibitor) supplemented with 1% benzonase and 0.5% NP-40, and incubated for 30 min on ice. Cell debris was pelleted at 16,000 g 10 min at 4°C and the lysate was diluted with washing buffer to a final detergent percentage of 0.2% before incubation with 20 µl GFP-trap agarose (Chromotek) for 2 h at 4°C under constant rotation. The beads were washed five times with washing buffer and eluted with 2x LDS sample buffer containing reducing agent.

Protein purification

BL21 *E. coli* bacteria were transformed with a pGEX-6P-1 plasmid encoding GST-ataxin-3. Overnight cultures of individual colonies were grown at 37°C in the presence of 100 µg/ml ampicillin. Part of the overnight culture was used to inoculate 500 ml LB with 100 µg/ml ampicillin. Bacteria were grown at 37°C until an OD600 of 0.6 and protein expression was induced by 1 mM IPTG for 3.5 h at 30°C. Bacteria were harvested (4000 rpm 15 min 4°C), resuspended in GST lysis buffer (50 mM HEPES pH 7.5, 500 mM NaCl, 10% glycerol, 1 mM MgCl2, 10 mM DTT) and lysed by sonication. The lysate was cleared (13.000 rpm 15 min 4°C) and incubated with equilibrated glutathione sepharose 4B beads (GE healthcare) overnight at 4°C under constant rotation. Beads were washed four times in GST lysis buffer and GST-ataxin-3 was eluted for 4 h at 4°C under constant rotation in elution buffer (50 mM Tris pH 7.5, 10 mM reduced glutathione, 10 mM DTT). To obtain untagged ataxin-3, beads were washed three times in PreScission cleavage buffer (50 mM Tris pH 7.5, 150 mM NaCl, 1 mM EDTA, 1 mM DTT) and protein was eluted by ca 40 units PreScission protease (GE healthcare) for 4 h at 4°C under constant rotation. Purified protein was

frozen in liquid nitrogen and stored at -70°C. Key steps of the purification process were analyzed on a Coomassie gel.

Supplementary References

Bennardo N, Cheng A, Huang N, Stark JM (2008) Alternative-NHEJ is a mechanistically distinct pathway of mammalian chromosome break repair. *PLoS Genet* **4:** e1000110

Boeddrich A, Gaumer S, Haacke A, Tzvetkov N, Albrecht M, Evert BO, Muller EC, Lurz R, Breuer P, Schugardt N, Plassmann S, Xu K, Warrick JM, Suopanki J, Wullner U, Frank R, Hartl UF, Bonini NM, Wanker EE (2006) An arginine/lysine-rich motif is crucial for VCP/p97-mediated modulation of ataxin-3 fibrillogenesis. *EMBO J* **25:** 1547-1558

Galanty Y, Belotserkovskaya R, Coates J, Jackson SP (2012) RNF4, a SUMO-targeted ubiquitin E3 ligase, promotes DNA double-strand break repair. *Genes Dev* **26:** 1179-1195

Mailand N, Bekker-Jensen S, Faustrup H, Melander F, Bartek J, Lukas C, Lukas J (2007) RNF8 ubiquitylates histones at DNA double-strand breaks and promotes assembly of repair proteins. *Cell* **131:** 887-900

Pierce AJ, Johnson RD, Thompson LH, Jasin M (1999) XRCC3 promotes homology-directed repair of DNA damage in mammalian cells. *Genes Dev* **13:** 2633-2638

Sowa ME, Bennett EJ, Gygi SP, Harper JW (2009) Defining the human deubiquitinating enzyme interaction landscape. *Cell* **138:** 389-403

Tang J, Cho NW, Cui G, Manion EM, Shanbhag NM, Botuyan MV, Mer G, Greenberg RA (2013) Acetylation limits 53BP1 association with damaged chromatin to promote homologous recombination. *Nat Struct Mol Biol* **20:** 317-325

Todi SV, Winborn BJ, Scaglione KM, Blount JR, Travis SM, Paulson HL (2009) Ubiquitination directly enhances activity of the deubiquitinating enzyme ataxin-3. *EMBO J* **28:** 372-382
